# Three-dimensional electroanatomical mapping guidelines for the selection of pacing site to achieve cardiac resynchronization therapy

**DOI:** 10.3389/fcvm.2022.843969

**Published:** 2022-09-30

**Authors:** Bao-Tong Hua, Li-Jin Pu, Xin Tian, Wen-Juan Song, Hao Li, Chao Wang, Xiao-Xia Shao, Rui Li, Shu-Min Li, Zhi-Xuan Li, Jun-Hua Zou, Ling Zhao, Jing Wang

**Affiliations:** ^1^Department of Geriatric Cardiology, The First Affiliated Hospital of Kunming Medical University, Kunming, China; ^2^Department of Cardiology, The First Affiliated Hospital of Kunming Medical University, Kunming, China

**Keywords:** three-dimensional electroanatomical mapping, left bundle branch area pacing, coronary venous pacing, cardiac resynchronization therapy (CRT), heart failure

## Abstract

**Objectives:**

We aimed to evaluate the feasibility of left ventricular electroanatomical mapping to choose between left bundle branch area pacing (LBBAP) or coronary venous pacing (CVP).

**Background:**

There are several ways to achieve left ventricular activation in cardiac resynchronization therapy (CRT): LBBAP and CVP are two possible methods of delivering CRT. However, the criteria for choosing the best approach remains unknown.

**Methods:**

A total of 71 patients with heart failure, reduced ejection fraction, and left bundle branch block (LBBB) were recruited, of which 38 patients underwent the three-dimensional electroanatomical mapping of the left ventricle to accurately assess whether the left bundle branch was blocked and the block level, while the remaining 33 patients were not mapped. Patients with true LBBB achieved CRT by LBBAP, while patients with pseudo-LBBB achieved CRT by CVP. After a mean follow-up of 6 months and 1 year, the QRS duration and transthoracic echocardiography, including mechanical synchrony indices, were evaluated.

**Results:**

Twenty-five patients with true LBBB received LBBAP, while 13 without true LBBB received CVP. Seventeen patients received LBBAP, and 16 patients received CVP without mapping. Paced QRS duration after the implantation of LBBAP and CVP was significantly narrower in the mapping subgroup compared to the non-mapping subgroup. A significant increase in post-implantation left ventricular ejection fraction was observed in patients with LBBAP or CVP, and the mapping subgroup were better than the non-mapping subgroup. After a 12-month follow-up, atrioventricular, intraventricular, and biventricular synchronization were significantly improved in the mapping subgroup compared to non-mapping groups in both LBBAP and CVP.

**Conclusion:**

In our study, three-dimensional electroanatomical mapping was used to choose LBBAP or CVP for heart failure patients, which proved feasible, with better cardiac resynchronization in the long-term follow-up. Therefore, three-dimensional electroanatomical mapping before CRT appears to be a reliable method for heart failure patients with LBBB who are indicated for CRT.

## Graphical Abstract

This study shows that three-dimensional electroanatomical mapping, with better cardiac resynchronization, provides informed guidance to choose between LBBAP or CVP for patients with heart failure. 
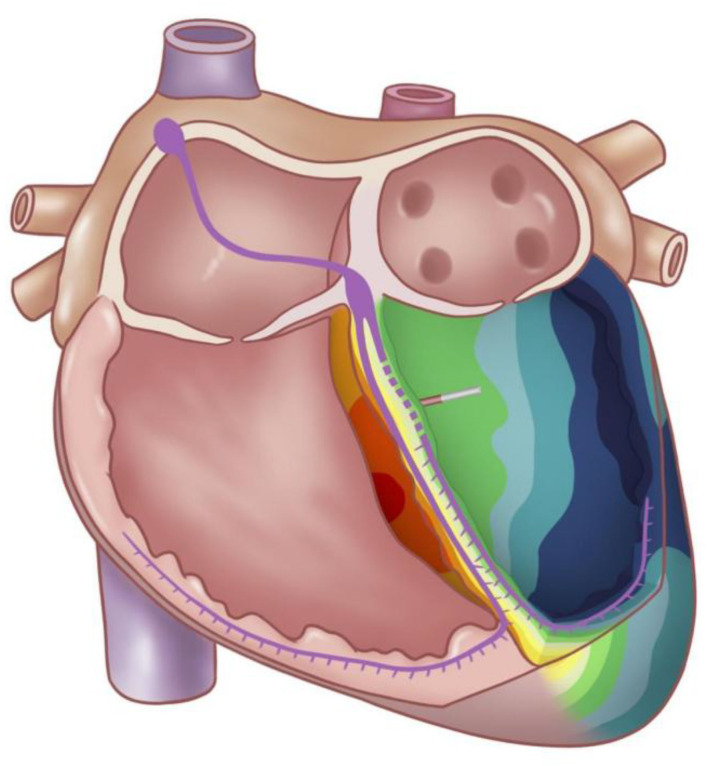

## Introduction

Cardiac resynchronization therapy (CRT) remains an important therapy for heart failure patients with biventricular desynchronization (1–3). There are currently several ways to achieve left ventricular activation in CRT, of which coronary venous pacing (CVP) and left bundle branch area pacing (LBBAP) are two possible methods of delivering CRT (4, 5). Although there is a non-response rate of up to 30%, significant sound evidence demonstrates that traditional cardiac resynchronization therapy (coronary venous pacing to the left ventricle) can significantly improve major adverse cardiovascular events in patients with heart failure (6). In recent years, the His-Purkinje system pacing has emerged, especially for the treatment of the left bundle branch area pacing (LBBAP) for cardiac resynchronization. Although LBBAP is generally considered to be a second-line strategy to BiV pacing, as its benefits over conventional CRT have not been demonstrated in randomized trials, some studies have shown that LBBAP is effective and safe in clinical trials (7–9). However, for patients with heart failure and a left bundle branch block (LBBB), there is currently no research that demonstrates which criteria should be used to choose left ventricular pacing to achieve CRT. Earlier studies suggested that patients with heart failure and LBBB can be divided into two categories: one is a true left bundle branch block (including slow conduction), and the other is that the conduction of the left bundle branch is generally normal while the left ventricular local Purkins or myocardial conduction is delayed and lead to the ECG features of LBBB (10). Through the three-dimensional electroanatomical mapping of the left ventricle, it is possible to accurately assess whether the left bundle branch is blocked and the block level (11). This study hypothesizes that, first, for patients with a true left bundle branch block, CRT can be achieved through the left bundle branch regional pacing. For patients with heart failure whose left bundle branch conduction is normal but the ECG shows LBBB, classical CRT to implant the left ventricular electrode in the lateral cardiac vein can be achieved. Second, the X image of the tip of the mapping catheter in the left bundle branch area or the latest activation area of the left ventricle can be used as a reference for the implantation of the left ventricular electrode, thereby facilitating CRT surgery.

## Methods

### Study population

**Figure 1:**
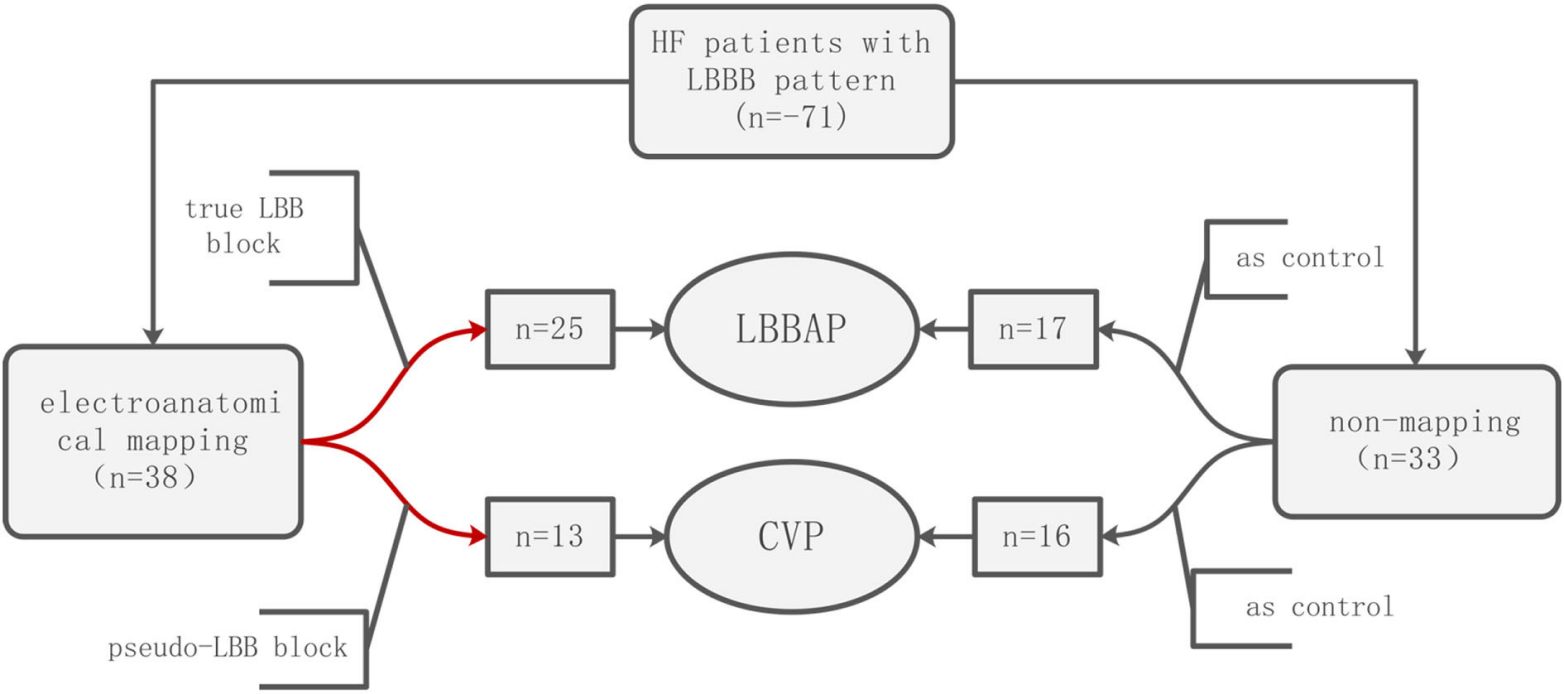
Patient flow.

This observational study recruited 71 consecutive patients with heart failure (HF) having reduced left ventricular ejection fraction (LVEF) and LBBB who had indications for CRT (Figure 1). Inclusion criteria for the study were LBBB with QRSd > 130 ms, LVEF <35%, and corresponding to the New York Heart Association functional class II to IV. All patients received standard medical treatment for at least 3 months before the implantation of the device. Twelve-lead electrocardiography (ECG) confirmed LBBB in all patients as defined by the American Heart Association, the American College of Cardiology Foundation, and the Heart Rhythm Society in 2009. Patients who could not give consent or were clinically unstable were excluded. All participants provided written informed consent. The study was approved by the Ethics Review Board of the First Affiliated Hospital of Kunming Medical University.

### Three-dimensional electroanatomical maps of the LV

A right femoral artery puncture and intubation was performed using the Seldinger method with an 8-Fr arterial sheath, and 35 units of unfractionated heparin per kilogram was administered. A mapping catheter (THERMOCOOL, 4 mm tip, Biosense Webster Inc., California, USA or mini-basket array catheter, Boston Scientific, Washington, DC, USA) was inserted from the sheath and advanced through the aortic valve with the J curve into the left ventricular (LV). Three-dimensional electroanatomical maps of the LV were reconstructed using a non-fluoroscopic navigation system (Fast Anatomical Mapping, CARTO 3^®^, version 6, Biosense Webster Inc. California USA) or electromagnetic navigation system (Rhythmia, Boston Scientific, Washington, DC, USA). First, activation mapping under sinus rhythm was performed to clarify the electroanatomical activation sequence of the left ventricle, especially the earliest and latest activation parts. Second, the potential of the His left bundle branch was mapped from the bottom to the apex of the left ventricular septum.

**Figure 2:**
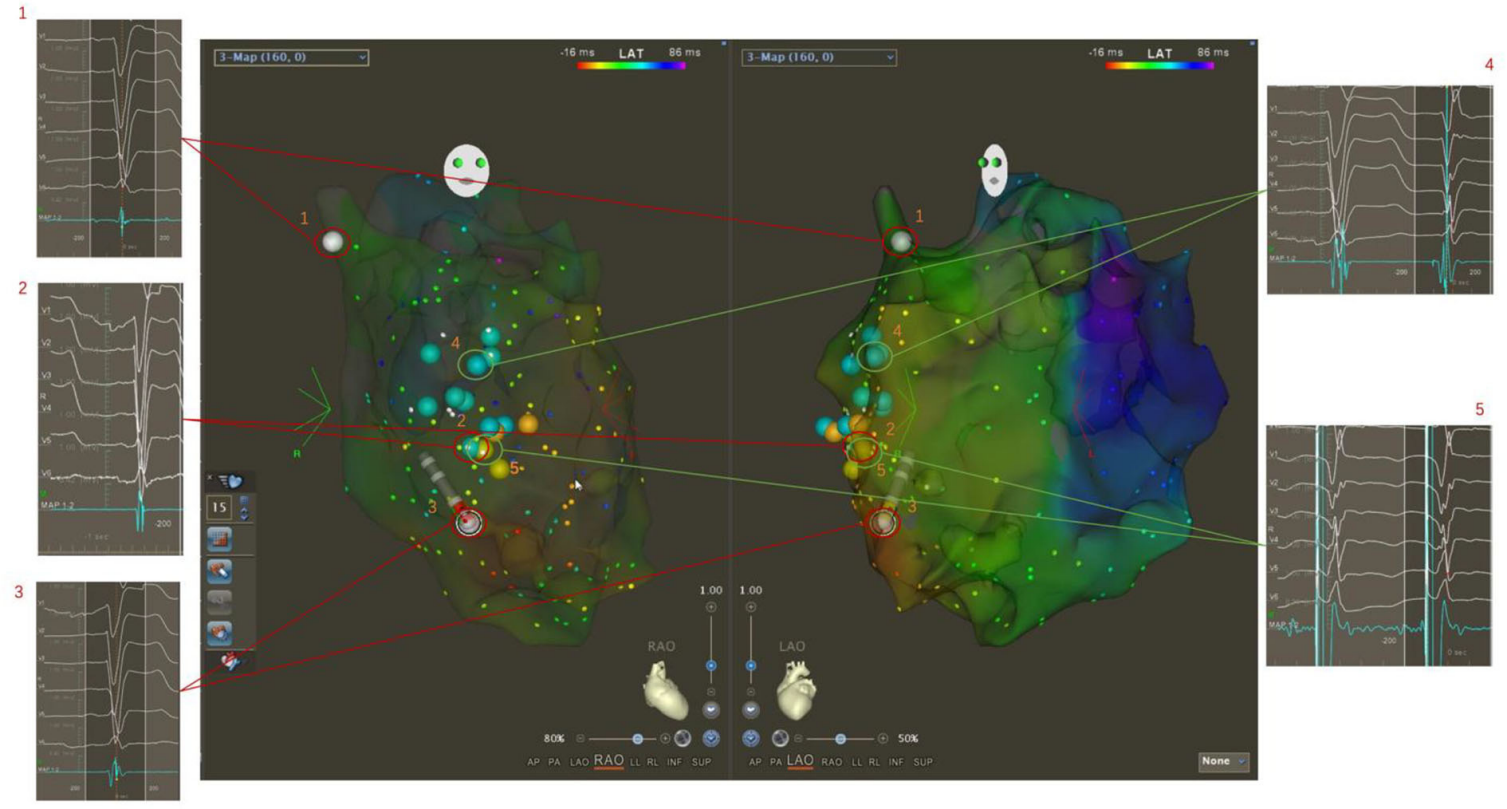
Three-dimensional electroanatomical mapping showing delayed conduction of the left bundle branch. The potentials of His and the left bundle branch were not mapped from the bottom to the apex of the left ventricular septum (1, 2, 3). The occasional mechanical stimulation in the left bundle branch area increased the potential and induced the subsequent narrow premature ventricular contraction (4). Then, electrical stimulation was performed in the upper middle area of the left bundle branch, and a QR or rSR morphology in the surface lead V1 was seen, which indicated there was a left bundle branch block or slow conduction. In the next step, LBBAP was planned (5).

If the obvious potential was not mapped in the left bundle branch area, the mechanical stimulation in this area could occasionally improve the left bundle branch potential and induce subsequent premature ventricular contraction. Then, electrical stimulation was applied in the upper-middle area of the left bundle branch, and a QR or rSR morphology in surface lead V1 was observed, indicating there was a left bundle branch block or slow conduction. In the next step, LBBAP was planned, and the X-ray image of the location of the mapping catheter tip was recorded to guide the placement of the left bundle branch area electrode (Figure 2).

**Figure 3:**
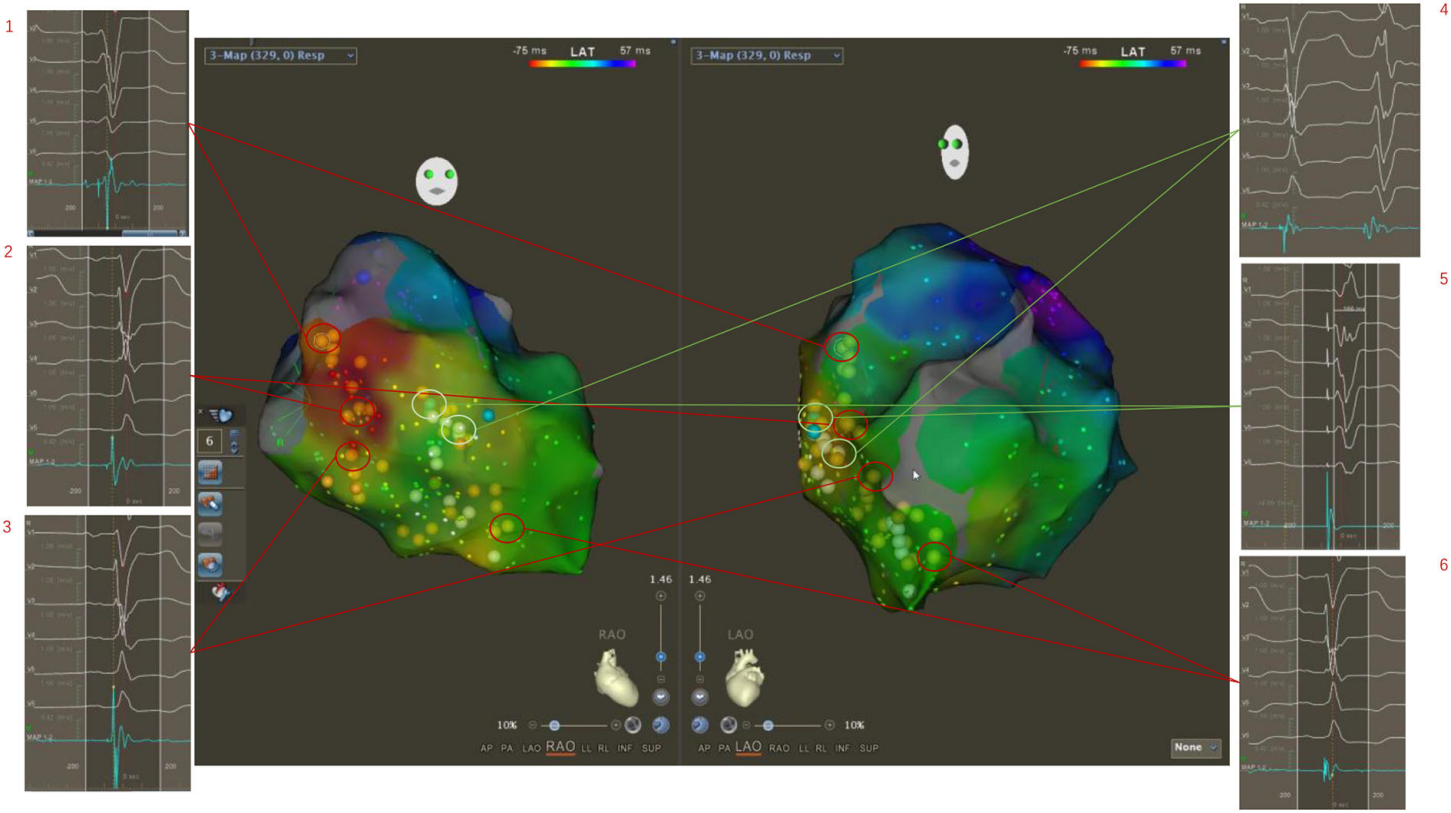
Three-dimensional electroanatomical mapping showed normal conduction of the left bundle branch although ECG showed LBBB pattern. The potentials of His and the left bundle branch were mapped from the bottom to the apex of the left ventricular septum (1, 2, 3, 6). The occasional mechanical stimulation in the left bundle branch area induced the subsequent wide premature ventricular contraction (4). Then, the electrical stimulation was performed in the upper middle area of the left bundle branch, and a wide rSR morphology in surface lead V1 was seen, which indicated the left bundle branch was normal (5). Classical CRT with coronary venous pacing was planned.

If the potential was mapped in the entire left bundle branch area, it indicated the patient's left bundle branch conduction was normal. Classical CRT with coronary venous pacing was planned, and the mapping catheter tip was placed on the latest activation part of the left ventricular activation; the X-ray image recorded the placement of the coronary venous electrode (Figure 3).

### Implantation procedure of CRT

Venous access was obtained *via* the left axillary vein for all patients. The atrial active fixed electrode was placed in the right atrial appendage. The right ventricular defibrillator electrode was first placed in the apex of the right ventricle.

For the patient with LBBAP-CRT, the pacing lead (model 3830, 69 cm, Medtronic Inc., Minneapolis, MN, USA) was inserted through a fixed-curve sheath (C315 His, Medtronic Inc.). An intracardiac electrogram was recorded from the lead tip by using the electrophysiological recording system (Bard Electrophysiology Lab System, MA, USA). The tip of the mapping catheter placed in the area of the left bundle branch in the left ventricle was recorded at the right anterior oblique (RAO) 30° position and left anterior oblique (LAO) 45° as references. The sheath and lead tip were advanced to the right ventricular septum that was directly opposite the mapping catheter tip and subsequently rotated in a counterclockwise fashion so the lead tip was in a perpendicular orientation to the interventricular septum (IVS). A “W”-shaped pacing morphology in surface lead V1 was observed at this location. As the lead tip was gradually screwed into the IVS, a rightward shift of the second notch in the “W”-shaped pacing morphology could be observed. The lead tip was in the final position after a QR or rSR morphology in surface lead V1 was achieved.

**Figure 4:**
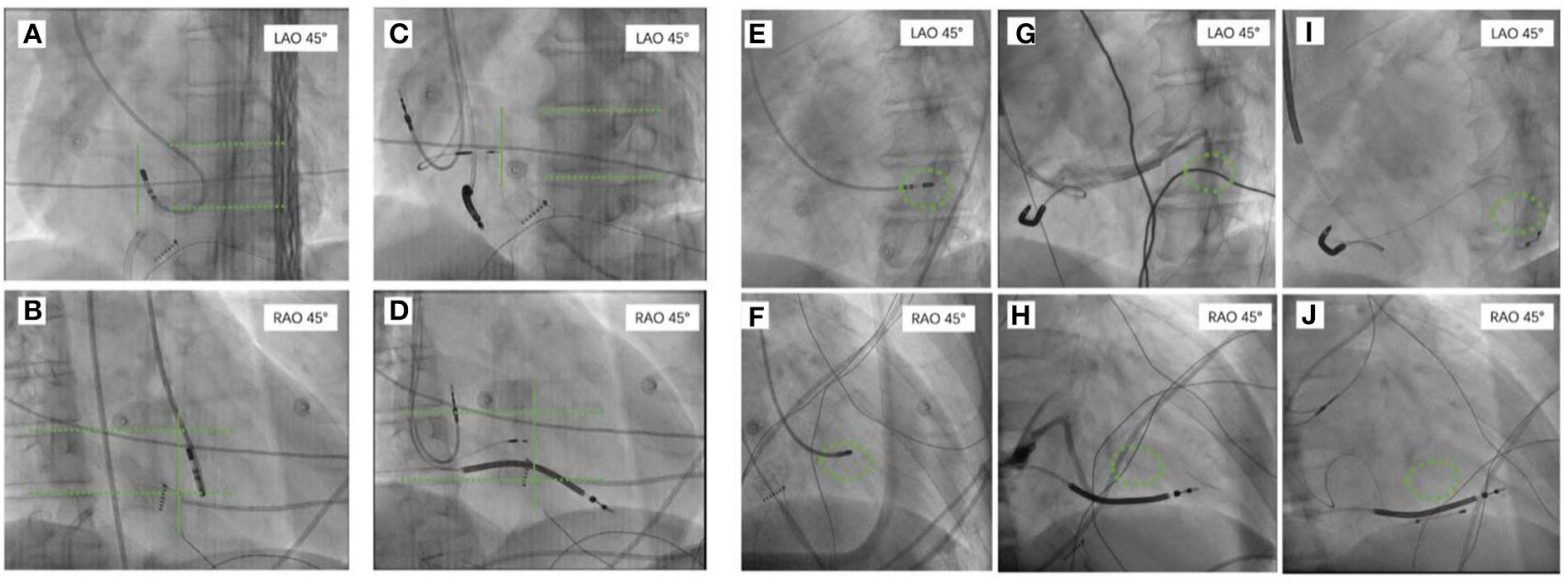
The tip of the mapping catheter under X-ray recorded at the right anterior oblique (RAO) 30° position and left anterior oblique (LAO) 45°. For patients who were planning to undergo LBBAP, the tip of the mapping catheter at the area of the left bundle branch in the left ventricle was recorded at RAO 30° and LAO 45° as references **(A,B)**. The 3,830 electrode was implanted on the right ventricular septum surface corresponding to the tip of the ablation catheter at RAO 30° and LAO 45° **(C,D)**. For patients who were planning to undergo CVP, the tip of the mapping catheter that was placed on the intima surface of the last activation of the left ventricle was recorded at RAO 30° and LAO 45° as references **(E,F)**. Coronary venography was performed to identify the cardiac vein **(G,H)** closest to the tip of the ablation catheter as the target for implantation of the left ventricular electrode **(I,J)**. RAO, the right anterior oblique; LAO, the left anterior oblique; CVP, coronary venous pacing; LBBAP, left bundle branch area pacing.

For patients with CVP-CRT, the tip of the mapping catheter at the area of the last activation in the left ventricle was recorded at the RAO 30° and LAO 45° positions as references. After retrograde angiography of the coronary sinus, the lateral veins or posterolateral veins that were the closest to the tip of the mapping catheter were used to target the blood vessel implanted with left ventricular lead (Figure 4).

### Programming of devices

A pacemaker was programmed to close the pacing function of the right ventricular pacing electrode but maintain the defibrillation function. The left ventricular lead pacing was gradually prolonged until the intracardiac electrogram showed atrial sensing–ventricular sensing. The atrial-ventricular delay (AVD) was shortened by 10-ms steps, and the ECG QRS duration was the narrowest. The corresponding AVD was optimized when the ECG QRS duration was the narrowest.

### Cardiac electrical synchrony evaluation

Cardiac electrical synchrony was assessed using the QRS duration of a 12-lead surface ECG. The surface ECG was obtained before and after the implantation. The QRS duration was measured from the onset of the intrinsic or paced QRS to the end of the QRS complex in all 12 leads. The left ventricular electrical synchrony was assessed using the LV activation time (LVAT), which was estimated by measuring the time from the intracardiac pacing spike to the R-wave peak of the QRS complex in lead V5 and V6. The widest-paced QRS duration and the wider LVAT were adopted for analyses. Two independent, experienced ECG specialists, blinded to the study, measured these two parameters.

### Cardiac mechanical synchrony evaluation

A Vivid E9 Doppler echocardiography (GE, USA) with M5S and 4V probes was used, with an emission frequency of 2.5 MHZ. The echocardiography examination was performed by the same echocardiologist, who was blinded to the study groups. The following indicators before and after the CRT operations were measured in the patients: the left ventricular ejection fraction (LVEF); the atrioventricular synchronous index: EA peak distance (E/A pd); the interventricular synchronous index: interventricular mechanical delay (IVMD); and the left ventricle synchronous index: standard deviation of Ts of 12 LV segments (Ts-SD12).

### Follow-up

The NYHA classifications were measured. Adverse events during the follow-up were recorded, including rehospitalization due to heart failure or death of the subjects, and the medical expenses essential for the mechanical treatment of chronic heart failure for the patients in the study group were also accurately recorded.

All patients underwent transthoracic echocardiography performed by an experienced specialist who was blinded to the study at the baseline as well as the 6th-month and 12-month follow-ups. The echocardiac measurement indicators, mentioned above, were recorded. Lead parameters, including R-wave amplitudes, capture thresholds, and pacing impedances were measured 1 week after the implantation.

### Statistical analysis

The continuous variables were presented as means ± standard deviations (SDs), and categorical variables were expressed as frequencies and proportions. Chi-square tests were used to compare categorical variables between the two subgroups. If variables were normally distributed, the parametric test (*t*-test) was adopted; if not, the non-parametric test (Mann–Whitney *U*-test) was used to compare numeric variables. Data at the baseline and follow-ups were compared by paired Wilcoxon signed rank tests. All statistical analyses were performed using SPSS 21.0 (IBM Corp, Armonk, NY, USA). The *P*-value statistical significance threshold was 0.05, two-tailed.

## Results

### Patients

A total of 71 patients were analyzed in this study, and 38 patients underwent three-dimensional mapping, of which 25 patients received left bundle branch area pacing LBBAP, and 13 patients received coronary venous pacing CVP; 33 patients did not receive mapping, of which 17 patients received left bundle branch area pacing LBBAP, and 16 patients received coronary venous pacing CVP. The baseline characteristics of the patients are summarized in Table 1. In the LBBAP group, 71.4% were diagnosed with dilated cardiomyopathy, and 14.2% were diagnosed with ischemic cardiomyopathy. Of the patients with no mapping, 12 were diagnosed with dilated cardiomyopathy, and two were diagnosed with ischemic cardiomyopathy. In the CVP group, 93.1% were diagnosed with dilated cardiomyopathy, and 3.4% were diagnosed with ischemic cardiomyopathy. Baseline age, gender, etiology, echocardiographic measurements, electrocardiogram parameters, and other items were not significantly different between the LBBAP and CVP subgroups.

**Table 1:** Baseline characteristics of patients analyzed (*n* = 71).

	**LBBAP**			**CVP**		
	**Mapping (*n* = 25)**	**No mapping (*n* = 17)**	***p*-values**	**Mapping (*n* = 13)**	**No mapping (*n* = 16)**	***p*-values**
Age, year	65.84 (10.45)	63.18 (8.85)	0.4	62.08 (11.49)	63.13 (14.41)	0.833
Male	11	13	0.06	10	10	0.45
**Diagnosis**						
DCM	18	12	0.78	11	16	0.10
Ischemic cardiomyopathy	4	2		1	0	
Other^*^	3	3		1	0	
Atrial fibrillation	3	3	0.67	0	3	0.09
Previous MI	6	2	0.07	1	1	1.00
**NYHA functional class**						
III and above	20	9	0.14	9	8	0.71
LVEF, %	31.16 (8.23)	28.24 (7.40)	0.25	30.62 (4.25)	27.31 (5.07)	0.072
IVS thickness, mm	9.36 (1.78)	9.06 (2.28)	0.63	9.62 (1.89)	9.38 (1.93)	0.739
LVEDD, mm	71.32 (9.13)	66.53 (7.08)	0.08	69.62 (9.78)	69.69 (8.17)	0.983
AVVTI	19.47(6.84)	17.25 (4.74)	0.32	20.09 (4.85)	17.6 (4.10)	0.169
Ts-SD 12	145.57 (18.93)	144.38 (41.72)	0.91	144.09 (27.84)	141.53 (44.40)	0.868
IVMD	58.19 (28.69)	71.62 (28.94)	0.2	67 (29.23)	62.8 (30.48)	0.727
EA distance/RR interval	0.28 (0.08)	0.26 (0.07)	0.43	0.27 (0.05)	0.22 (0.07)	0.069
**Drug therapy**						
β-blocker	24	17	1	12	11	0.18
ACEI/ARB	14	10	1	6	6	1
Spironolactone	23	15	1	12	10	0.1
ARNI	8	9	0.21	7	10	0.7
PR interval, ms	180.96 (49.61)	183.82 (47.06)	0.85	182.92 (35.06)	165.44 (27.96)	0.146
QRS duration, ms	165.72 (17.34)	164.00 (18.20)	0.76	170.54 (29.82)	163.88 (24.35)	0.513
QRS notch width, ms	56.00 (8.57)	42.18 (8.96)	<0.001	41.69 (17.19)	42.19 (15.02)	0.935
**Type of CRT**						
CRT-D	18	15	0.19	8	15	0.1
CRT-P	5	1		4	1	
Double chamber	2	1		1	0	

^*^Other includes the following: alcoholic cardiomyopathy, hypertensive cardiomyopathy, and non-compaction of myocardium.

DCM, dilated cardiomyopathy; MI, myocardial infarction; NYHA, The New York Heart Association; LVEF, left ventricular ejection fraction; IVS, interventricular septum; LVEDD, left ventricular end-diastolic dimension; AVVTI, aortic valve velocity time integral; Ts-SD 12, standard deviation of 12-segment time to peak systolic velocity; IVMD, interventricular mechanical delay time; ARNI, angiotensin receptor neprilysin inhibitor; CRT-D, cardiac resynchronization therapy-defibrillation; CRT-P, cardiac resynchronization therapy-pacemaking.

### Electroanatomic mapping

Thirty-eight patients successfully performed LV electroanatomical mapping. Twenty-five patients had delayed conduction of the left bundle branch, of which nine cases were in the middle and upper part of the left bundle branch, and 16 cases were in the left-sided His fibers. No true complete block of the left bundle branch was found, which had slow conduction rather than an inability to conduct. In other words, the bundle branch potential that is usually obscured in the local V wave was advanced by electrical or mechanical stimulation, and we saw an induced premature ventricular contraction that had a QR or rSR morphology in surface lead V1. It showed that left bundle branch conduction was delayed, which was the next step for the inclusion of CRT with LBBAP. In 16 cases, the left bundle branch potential was completely mapped from the bottom of the heart to the apex, and it was always ahead of the local myocardial activation, suggesting the left bundle branch conduction was normal. The next step was to achieve CRT through CVP.

### Lead parameters

The lead parameters, including capture thresholds, pacing impedances, and R-wave amplitudes are provided in the Supplementary materials.

### Complications

One LBBAP mapped patient was diagnosed with hemopneumothorax about 4 h after successful surgery, and the patient underwent closed thoracic drainage and blood transfusion therapy. No patient with a loss of capture, lead removal, or late lead dislodgement was observed. Echocardiography showed the pacing lead was positioned at the sub-endocardium of IVS in all LBBAP patients.

### Electrical synchrony evaluation

**Figure 5:**
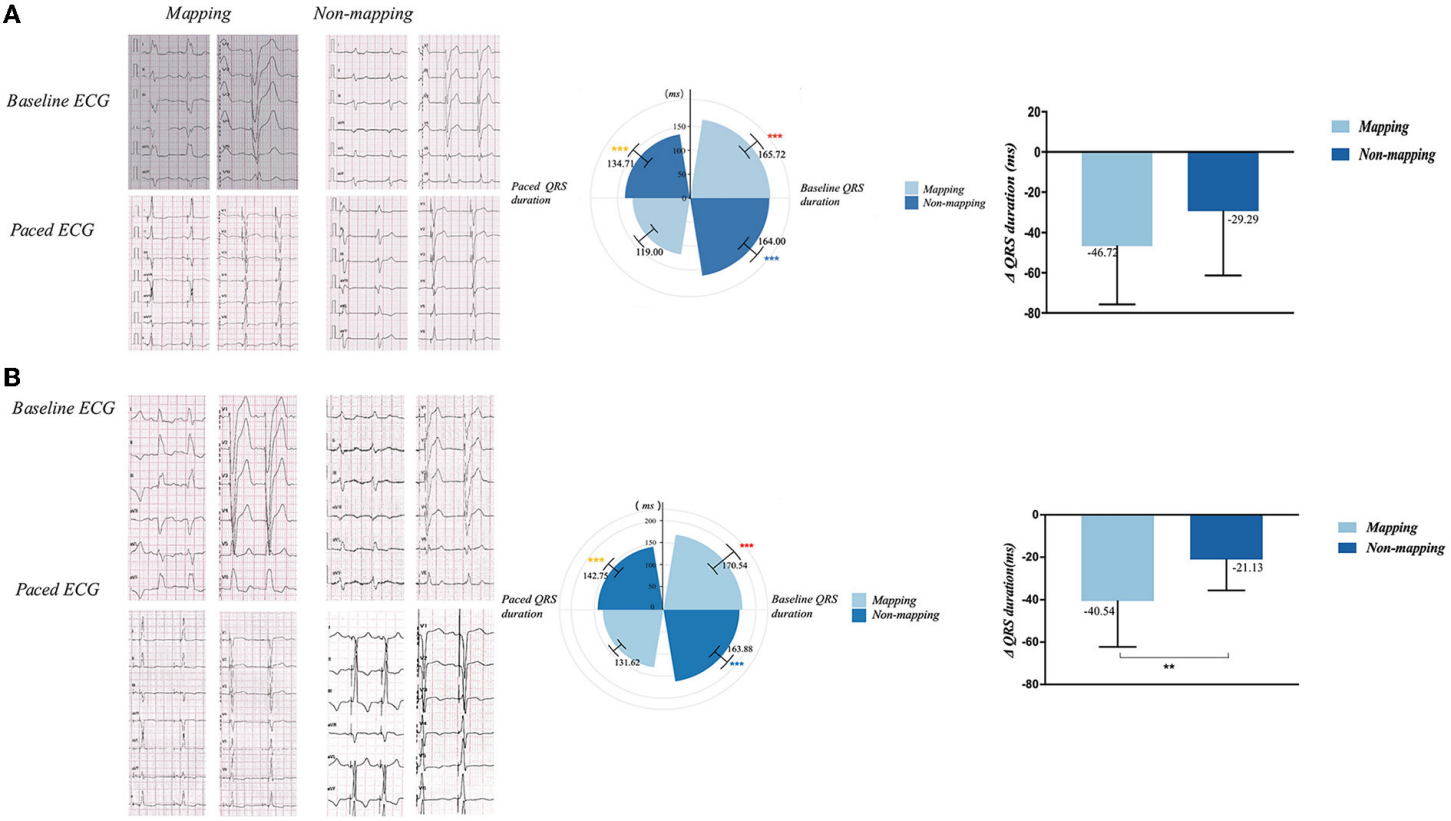
QRS duration and QRS duration variation of mapping and non-mapping group in LBBAP **(A)** and CVP **(B)** before and 1-week after the operation. ***p < 0.001 vs. paced QRS duration in mapping group; ***p < 0.001 vs. paced QRS duration in non-mapping group; ***p < 0.001 vs. paced QRS duration in mapping group; *p < 0.05; **p < 0.01; CVP, coronary venous pacing; LBBAP, Left bundle branch region pacing.

In the LBBAP group, paced QRS duration 1 week after the implantation was significantly narrower than the baseline in both the mapping (165.72 ± 17.34 ms vs. 119.00 ± 20.88 ms, *p* < 0.001) and no mapping subgroups (164.00 ± 18.20 ms vs. 134.71± 20.30 ms, *p* = 0.002). Furthermore, the paced QRS duration exhibited a significant difference between the mapping and no mapping subgroups (119.00 ± 20.88 ms vs. 134.71 ± 20.30 ms, *p* = 0.02). In the CVP group, paced QRS duration 1 week after the implantation was significantly narrower than the baseline in the mapping (170.54 ± 29.82 ms vs. 131.62 ± 11.67 ms, *p* = 0.001) and no mapping subgroups (163.88 ± 24.35 ms vs. 142.7 ± 14.74 ms, *p* < 0.001); paced QRS duration showed a considerable difference between the mapping and no mapping subgroups (131.62 ±11.67 ms vs. 142.7 ± 14.74 ms, *p* = 0.035). The reductive value of QRS duration from baseline to post-operation was significant only in the CVP mapping and non-mapping groups (−21.13 ± 14.58 vs. −40.54 ± 21.66, *p* = 0.008; see Figure 5).

### Mechanical synchrony evaluation

**Figure 6:**
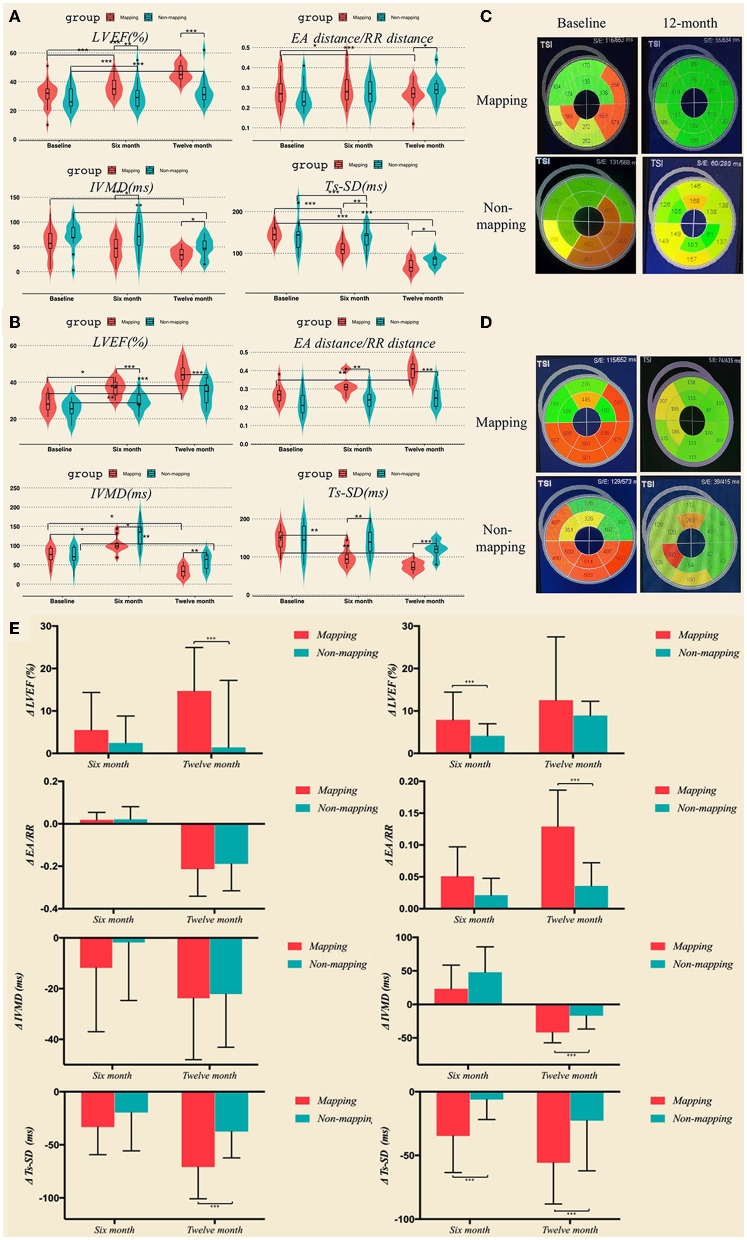
LVEF and synchronization parameters including EA distance/RR duration, IVMD, and Ts-SD 12 in LBBAP **(A)** and CVP **(B)** at baseline, 6 months, and 12 months after the operation measured by transthoracic echocardiography. **(C,D)** Bulls-eye view of real-time three-dimensional echocardiography at baseline and 12 months after the operation. **(E)** LVEF and synchronization parameters variation in LBBAP and CVP group at 6 months and 12 months. *p < 0.05; **p < 0.01; ***p < 0.001. CVP, coronary venous pacing; LBBAP, Left bundle branch region pacing.

In the LBBAP group, 6 months after undergoing CRT, the LVEF, intraventricular synchronization index Ts-SD12, and atrioventricular synchronization index EA/RR were significantly improved when compared to the baseline in the LBBAP mapping subgroup (LEVF 31.16 ± 8.23% vs. 37.64 ± 6.18%, *p* < 0.001; Ts-SD12 145.57 ms ± 18.93 vs. 111.38 ± 18.74 ms, *p* < 0.001; EA/RR 0.28 ± 0.08 vs. 0.30 ± 0.08, *p* = 0.026). In the LBBAP no mapping grouping, the LVEF and the intraventricular synchronization index Ts-SD12 significantly improved when compared to the baseline (LEVF 28.24 ± 7.40% vs. 32.71 ± 6.99%, *p* = 0.001; Ts-SD12 144.38 ± 4 1.72 ms vs. 131.62 ± 38.02 ms, *p* = 0.001). Between the two subgroups, there were significant differences in LEVF, IVMD, and Ts-SD12 (LVEF 37.64 ± 6.18% vs. 32.71 ± 6.99%, *p* = 0.009; IVMD 46.38 ± 22.67 ms vs. 69.77 ± 31.64 ms, *p* = 0.043; and Ts-SD12 111.38 ± 18.74 ms vs.131.62 ±38.02 ms, *p* = 0.007). In the 12 month follow-up, echocardiography parameters including LVEF, atrioventricular synchronization index EA/RR, biventricular synchronization index IVMD, and intraventricular synchronization index Ts-SD 12 were significantly improved when compared to the baseline data in the mapping subgroup (LEVF 31.16 ± 8.23% vs. 52.36 ± 6.05%, *p* < 0.001; IVMD 58.19 ± 28.69 ms vs. 39.90 ± 17.99 ms, *p* = 0.002; Ts-SD12 145.57 ± 18.93 ms vs. 121.24 ± 27.43 ms, *p* = 0.001; and EA/RR 0.28 ± 0.08 vs. 0.34 ± 0.10, *p* = 0.001). LVEF, IMVD, and Ts-SD12 significantly improved when compared to the baseline in the LBBAP no mapping subgroup (LEVF 28.24 ± 7.40% vs. 40.60 ± 7.84%, *p* = 0.001; IVMD 71.62 ± 28.94 ms vs. 50.23 ± 21.43 ms, *p* = 0.008; and Ts-SD12 144.38 ± 41.72 ms vs. 123.62 ± 38.23 ms, *p* = 0.003). Furthermore, the LVEF, EA/RR, IVMD, and Ts-SD 12 exhibited a significant difference between the mapping and no mapping subgroups (LEVF 52.36 ± 6.05% vs. 40.60 ± 7.84%, *p* < 0.001; IVMD 39.90±17.99 ms vs. 50.23 ± 21.43 mm, *p* = 0.024; TSSD121.24 ± 27.43 ms vs.123.62 ± 38.23 ms, *p* = 0.029; and EA/RR 0.34 ± 0.10 vs. 0.29 ± 0.06, *p* = 0.014; Figures 6A,C and Supplementary Table 2).

Although in the CVP group, the mapping subgroup's LVEF, intraventricular synchronization index Ts-SD12, and IVMD were noticeably enhanced 6 months after CRT (LVEF 30.62 ± 4.25% vs. 35.77 ± 5.76%, *p* = 0.013; Ts-SD12 144.09 ± 27.84 ms vs. 130.82 ± 32.80 ms, *p* = 0.003; and IVMD 67.0 ± 29.23 ms vs. 55.72 ± 23.43 ms, *p* = 0.022). The LVEF and IVMD were significantly improved when compared to the baseline in the no mapping subgroup (LVEF 27.31 ± 5.07% vs. 29.06 ± 4.67%, *p* = 0.001; IVMD 62.80 ± 30.48 ms vs. 59.60 ± 28.76 ms, *p* = 0.001); and there was a great disparity in LVEF, EA/RR, IVMD, and Ts-SD 12 between the mapping and no mapping subgroups (LVEF 35.77 ± 5.76% vs. 29.06 ± 4.67%, *p* = 0.001; Ts-SD12 130.82 ± 32.80 ms vs. 137.27 ± 34.16 ms, *p* = 0.01; IVMD55.72 ± 23.43 ms vs. 59.60 ± 28.76 ms, *p* = 0.043; EA/RR 0.29 ± 0.04 vs. 0.22 ± 0.05, *p* = 0.002). At 12-month follow-up, the LVEF, EA/RR, IVMD, and Ts-SD 12 were observed to have significantly improved when compared to the baseline data in the mapping subgroup (LVEF 30.62 ± 4.25% vs. 39.92 ± 5.87%, *p* = 0.01; Ts-SD12 144.09 ± 27.84 ms vs. 127.0 ± 35.62 ms, *p* = 0.006; IVMD 67.0 ± 29.23 ms vs. 52.45 ± 24.17 ms, *p* = 0.016; EA/RR 0.27 ± 0.05 vs. 0.40 ± 0.05, *p* = 0.003); LVEF and IMVD were substantially improved when compared to the baseline in the LBBAP no mapping subgroup (LVEF 27.31 ± 5.07% vs. 33.88 ± 6.24%, *p* < 0.001; IVMD 62.8 ± 30.48 ms vs. 57.53 ± 20.81 ms, *p* = 0.004). In addition, the LVEF, EA/RR, IVMD, and Ts-SD 12 exhibited a significant difference between the mapping and no mapping subgroups (LVEF 39.92 ± 5.87% vs. 33.88 ± 6.24%, *p* < 0.001; Ts-SD12 127.0 ± 35.62 ms vs. 119.73 ± 21.43 ms, *p* < 0.001; IVMD 52.45 ± 24.17 ms vs. 57.53 ±20.81 ms, *p* = 0.006; EA/RR 0.40 ± 0.05 vs. 0.23 ± 0.70, *p* < 0.001; Figures 6B,D and Supplementary Table 3).

In the LBBAP group, patients who received mapping before the operation showed significantly greater improvement in LVEF and Ts-SD 12 at 1-year follow-up, compared to those who underwent CRT without mapping (LVEF: 1.41 ± 15.81 vs. 14.72 ± 10.22, *p* = 0.002; Ts-SD12: −37.54 ± 24.77 vs. −70.90 ± 29.94, *p* = 0.002; Figure 6E left column). With regard to CVP, both the LVEF at 6-month follow-up and synchronization indicators at 12-month follow-up exhibited significantly greater absolute variation in those whose left ventricular was mapped before CRT, as clarified in Figure 6E (right column).

### Clinical outcome

All LBBP patients survived with greater improvement in cardiac function during a mean follow-up of 11.5 ± 3.3 months in the LBBAP group. During the clinical follow-up, NYHA classification was found to be improved compared to the baseline in both the mapping and no mapping subgroups of the LBBAP group and CVP group. No statistically significant difference was observed in clinical indicators between the two groups after the operation.

## Discussion

At present, there are several ways to correct the CLBBB to achieve the CRT. The classical implantation of the left ventricular electrode through the lateral cardiac vein and the implantation of the left ventricular electrode in the left bundle branch are two possible methods of delivering CRT. Left bundle branch area pacing is emerging recently, while considered to be a second-line strategy to BiV pacing, as its benefits over conventional CRT have not been demonstrated in randomized trials. There is no research on the criteria for choosing between these two methods for patients with heart failure and CLBBB who conform to the CRT indications. A new study demonstrated that LBBP-CRT had better electromechanical resynchronization and higher clinical and echocardiographic response than BVP-CRT in HFrEF patients with LBBB (12).

This study first demonstrated that three-dimensional electroanatomical mapping of the left ventricle can be used to determine the conduction in the left bundle branch and the level of blockage. If the left bundle branch is blocked or slowed, CRT is achieved by pacing in the left bundle branch area; if the left bundle branch conduction is normal, CRT is achieved by the traditional lateral cardiac vein pacing. Second, the X-imaging of the tip of the mapping catheter in the left bundle branch area or the latest activated area of the left ventricle can be used as an important reference for the implantation of the left ventricular electrode into the target point or vessel during CRT implantation, which simplifies the surgical procedure.

### Different subtypes of left bundle branch block

Left bundle branch block has received increased attention in past last decade. It has largely been associated with the implementation of the CRT and accumulative data demonstrate a considerably higher rate of response in patients with LBBB QRS morphology (13). Historically, wide (≥120 ms) QRS patterns with dominant S-waves in lead V1 have been aggregated into the broad categorization of an LBBB pattern. However, it is worth noting that these criteria were introduced in 1941 on a dog model and extrapolated to humans. The prevailing definition of the LBBB pattern was developed by the American Heart Association, the Electrocardiography and Arrhythmias Committee, the Council on Clinical Cardiology, the American College of Cardiology Foundation, and the Heart Rhythm Society (AHA/ACCF/HRS) in 2009. The LBBB pattern required a QRS ≥ 120 ms with a broad notched or slurred R-wave in leads I, aVL, V5, and V6 (14). In 2011, Strauss and colleagues proposed a cut-off of ≥140 ms in men and ≥130 ms in women as well as the requirements of a QS or RS in leads V1–V2 and mid-QRS notching or slurring in two or more of leads V1, V2, V5, V6, I, and aVL (15).

Tung et al. (10) commenced electrophysiology testing to delineate the activation patterns of the proximal left conduction system with multielectrode catheters in patients presenting for cardiac resynchronization. They reported that in patients with LBBB, the block was most often localized to the left-sided His fibers (46%). Less commonly, the LBBB was found distal to the His recording site (18%), at locations in which an atrial electrogram was not recorded. These locations were anatomically consistent with the block in the distal branching bundle or proximal left bundle-branch. The remainder (36%) of the patients with an LBBB pattern did not demonstrate a complete conduction block. Assessment of local ventricular electrograms showed an intact Purkinje activation and the QRS was wide, most likely because of conduction slowing more distally. Multiple ECG criteria have been assessed, but without using a “gold standard” of determination of whether the block was present (10).

Direct placement of the pacing electrode is difficult (16), there are three difficulties for doctors performing this operation: finding the His bundle potential, controlling the depth of rotation in the septum, and choosing other sites if the position is inappropriate. In this study, the left bundle branch area of the left ventricular septum could be paced directly through the mapping electrode, and offer an X-ray image as a location reference. Similarly, the activation of the left ventricle is well-represented by maps in CVP.

### Left univentricular pacing achieving CRT

To ensure a 100% biventricular capture, a conventional CRT used the short and fixed AV delay and an abandoned intrinsic activation from the right bundle branch (17). Activation from non-physiological biventricular pacing caused slow propagation and inverse conduction in the His-Purkinje system, resulting in an intraventricular pseudo-resynchronization (17, 18). In this study, a pacemaker was programmed to close the pacing function of the right ventricular pacing electrode while maintaining the defibrillation function. The left bundle branch area pacing without right ventricular pacing not only corrected LBBB but also generated a relatively normal pattern of ventricular activation, such that one activation wave was in front of the left bundle branch by pacing, while the other activation wave was from the intrinsic conduction of the right bundle branch (19). Coronary venous pacing advanced the last excited area of the left ventricle, which synchronized with the intrinsic activation from the left and right bundle branch. Synchronizing the LBBAP or CVP with intrinsic activation to achieve physical resynchronization can improve CRT efficacy (20).

### Efficacy of CRT guided by mapping

After the three-dimensional electroanatomical mapping, patients with complete left bundle branch block were chosen for LBBAP. We observed a significant reduction in QRS duration and an improvement in LVEF in the mapping subgroup of CVP and LBBAP, which suggested that implantation of CRT after three-dimensional electroanatomical mapping results in better cardiac resynchronization. For patients with a true left bundle branch block, CRT can be achieved through the left bundle branch regional pacing by correcting LBBB and restoring normal physiological LV activation.

### Limitations

Being a retrospective study, the main weakness is that patients were not randomized to mapping or non-mapping groups. Therefore, the homogeneity of the data is relatively poor and the level of evidence is not strong.

In this study, electroanatomical mapping of the left ventricular cavity was performed by puncturing the femoral artery and administering heparin (30 IU/Kg). After the mapping, protamine was given to neutralize the heparin (1 mg:100 IU), and the implantation of CRT was continued. Although it was not found in this study, the complications of vascular puncture and the risk of blood oozing from the pocket are theoretically increased during this step.

This study was a single-center prospective study, and the sample size was relatively small. Even though our study demonstrated novel criteria for choosing between CVP or LBBAP, which is safe and feasible, larger and randomized controlled trials should be conducted to verify its long-term safety and clinical benefits.

## Conclusion

For heart failure patients with LBBB who are indicated for CRT, left ventricular electroanatomical mapping before CRT, which determines whether the left bundle branch is blocked or not, is a safe, feasible way to choose between LBBAP or CVP. The benefit of the pre-procedural mapping is that patients with intact left bundle branch conduction (and slow distal conduction) are filtered out and do not receive LBBAP, as they are unlikely to benefit. In addition, the X-ray image of the mapping catheter tip at the left bundle branch area, or the latest activation area of LV can provide a positioning reference for the implantation of the left ventricular electrode into the target area.

## Perspectives

### What is known?

There are currently two ways to achieve left ventricular activation in CRT. One is to place a left ventricular electrode in the coronary venous pacing (CVP), and the other is to place a left ventricular electrode in the left bundle branch area.

### What is new?

For patients with heart failure and left bundle branch block (LBBB), there is currently no research that recommends which criteria should be used to choose left ventricular pacing to achieve CRT.

### What is next?

Further studies with larger and randomized controlled trials should be conducted to verify the findings of this study and identify the long-term safety and clinical benefits of three-dimensional electroanatomical mapping-guided CRT.

## Data availability statement

The raw data supporting the conclusions of this article will be made available by the authors, without undue reservation.

## Ethics statement

The studies involving human participants were reviewed and approved by the Ethics Review Board of the First Affiliated Hospital of Kunming Medical University. The patients/participants provided their written informed consent to participate in this study.

## Author contributions

JW and LZ designed and supervised the study. B-TH, JW, and L-JP performed CRT implantation and drafted this manuscript. XT performed statistical analyses. HL, W-JS, XT, CW, X-XS, RL, S-ML, Z-XL, and J-HZ followed the patients and optimized the parameters. All authors contributed to the article and approved the submitted version.

## Funding

This work was supported by the National Natural Science Foundation of China (81960069; PI: JW), Science and Technology Department of Yunnan Province [2019FE001(-139); PI: JW], the Yunnan Provincial Youth Top-notch Talent Support Program (RLQB20200009), and Yunnan Provincial High-level Health Technical Reserve Talents Support Program (H-2019051).

## Conflict of interest

The authors declare that the research was conducted in the absence of any commercial or financial relationships that could be construed as a potential conflict of interest.

## Publisher's note

All claims expressed in this article are solely those of the authors and do not necessarily represent those of their affiliated organizations, or those of the publisher, the editors and the reviewers. Any product that may be evaluated in this article, or claim that may be made by its manufacturer, is not guaranteed or endorsed by the publisher.

## Abbreviations

CRT: Cardiac resynchronization therapy
CVP: Coronary venous pacing
LBBAP: Left bundle branch area pacing
LBBB: Left bundle branch block
LVEF: Left ventricular ejection fraction
IVMD: Interventricular mechanical delay
Ts-SD12: Standard deviation of Ts of 12 LV segments
TTE: Transthoracic echocardiography.
