# Supplementary material for: Three-dimensional electroanatomical mapping guidelines for the selection of pacing site to achieve cardiac resynchronization therapy
Source: Front Cardiovasc Med. 2022 Sep 30;9:843969. doi: 10.3389/fcvm.2022.843969 (PMC9562822; doi:10.3389/fcvm.2022.843969)
Supplement: Supplementary file 1 [file Data_Sheet_1.pdf]

We did use two different mapping catheters for activation mapping on the left ventricular septal surface, but because of cost, most of our mapping patients used the Biosense ThermoCool catheter. Only 4 cases were used with the Boston Scientific Orion mini-basket catheter, and all belonged to the LBBP group. The figures below details the mapping results of this basket catheter, which is also consistent with the results of other LBBP mapping groups with the Biosense ThermoCool catheter.

The HIS potential was mapped in the region of the His bundle on the left ventricular septum

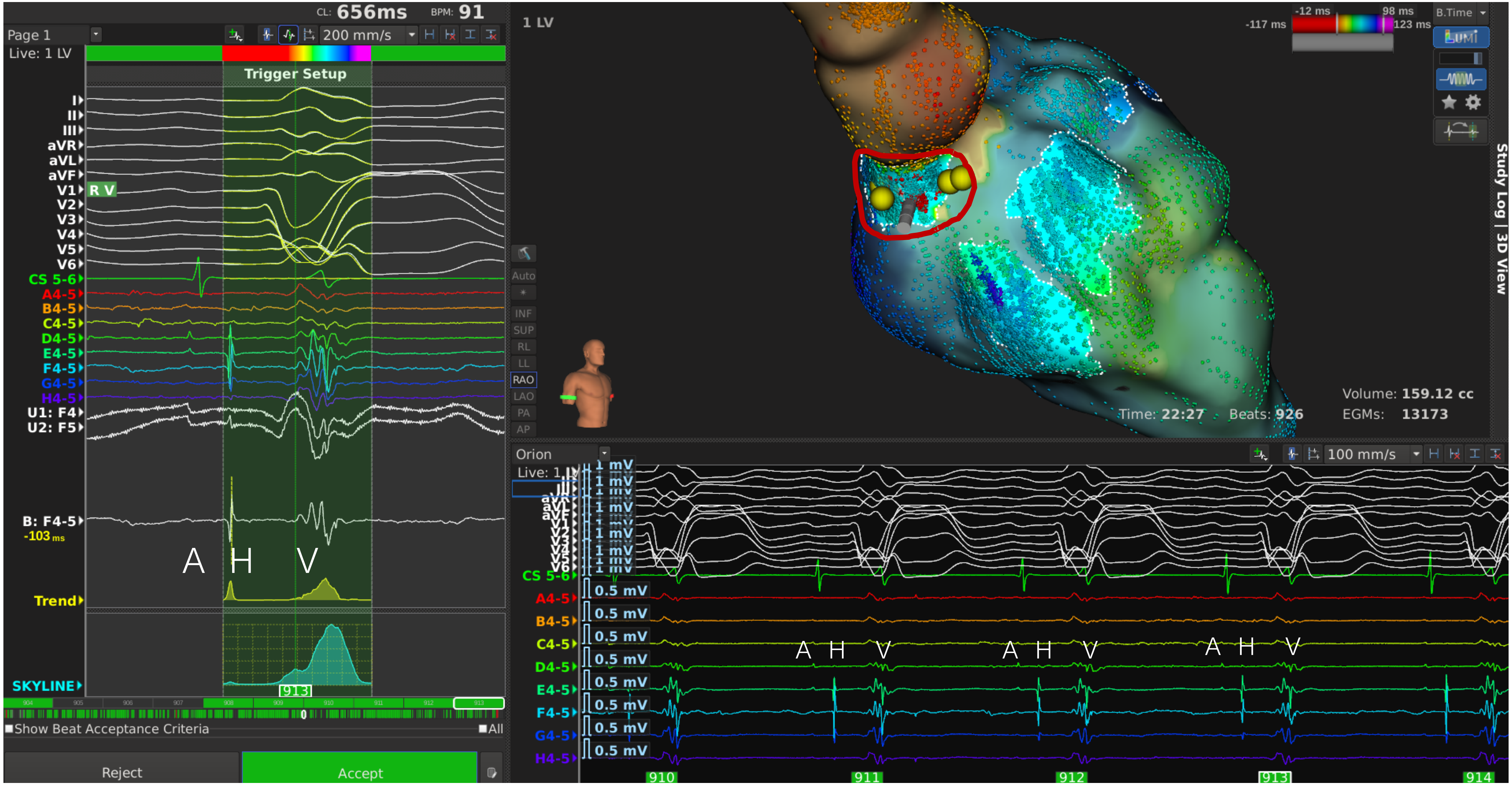

Although Ultra high-resolution mapping by Boston Scientific Orion mini-basket catheter was used along down the the left septum , there was no left bundle branch potential were identified before the local ventricular potential activation

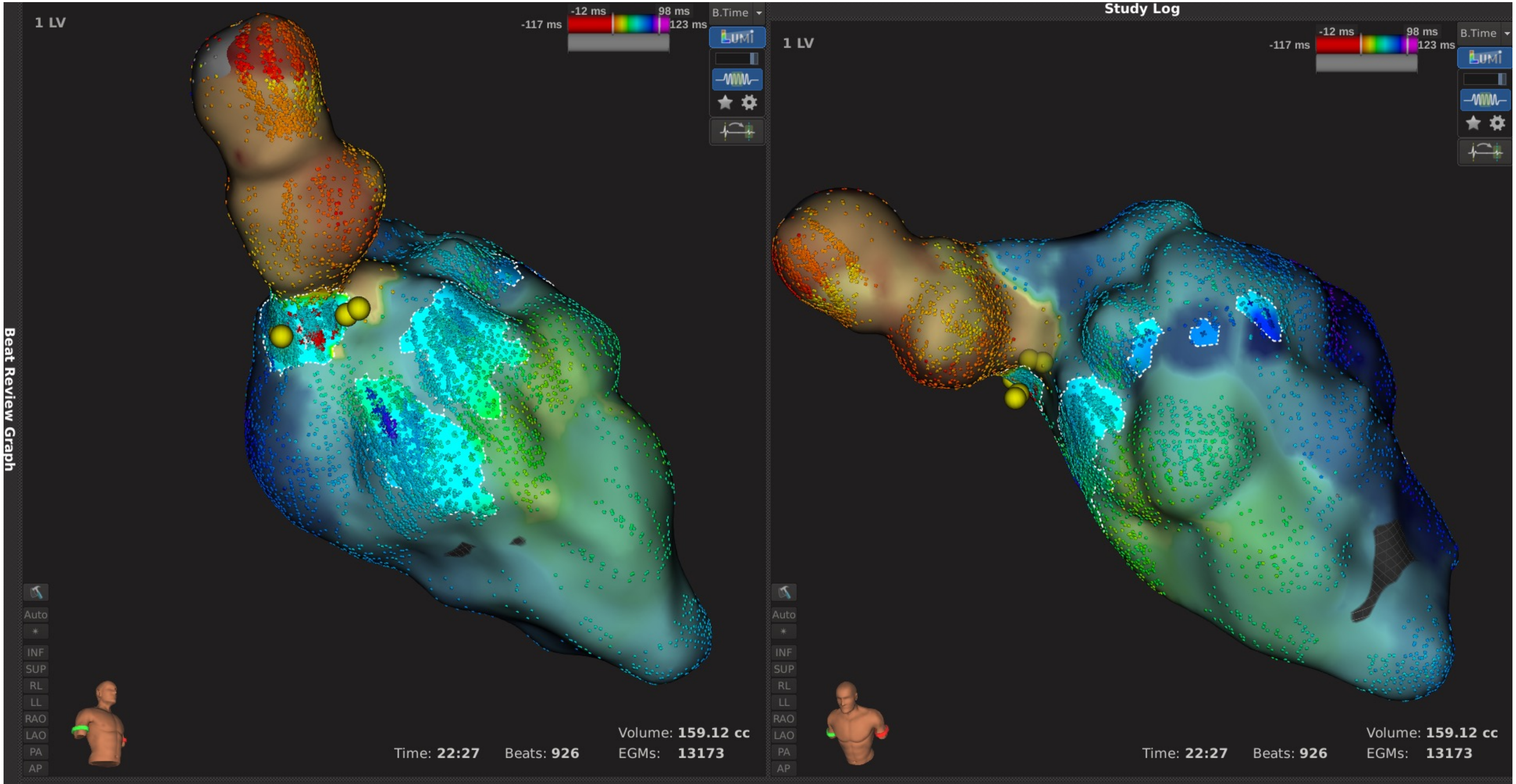

Careful analysis of the local potential in the left bundle branch region revealed that the left bundle branch potential was later than the local ventricular activation

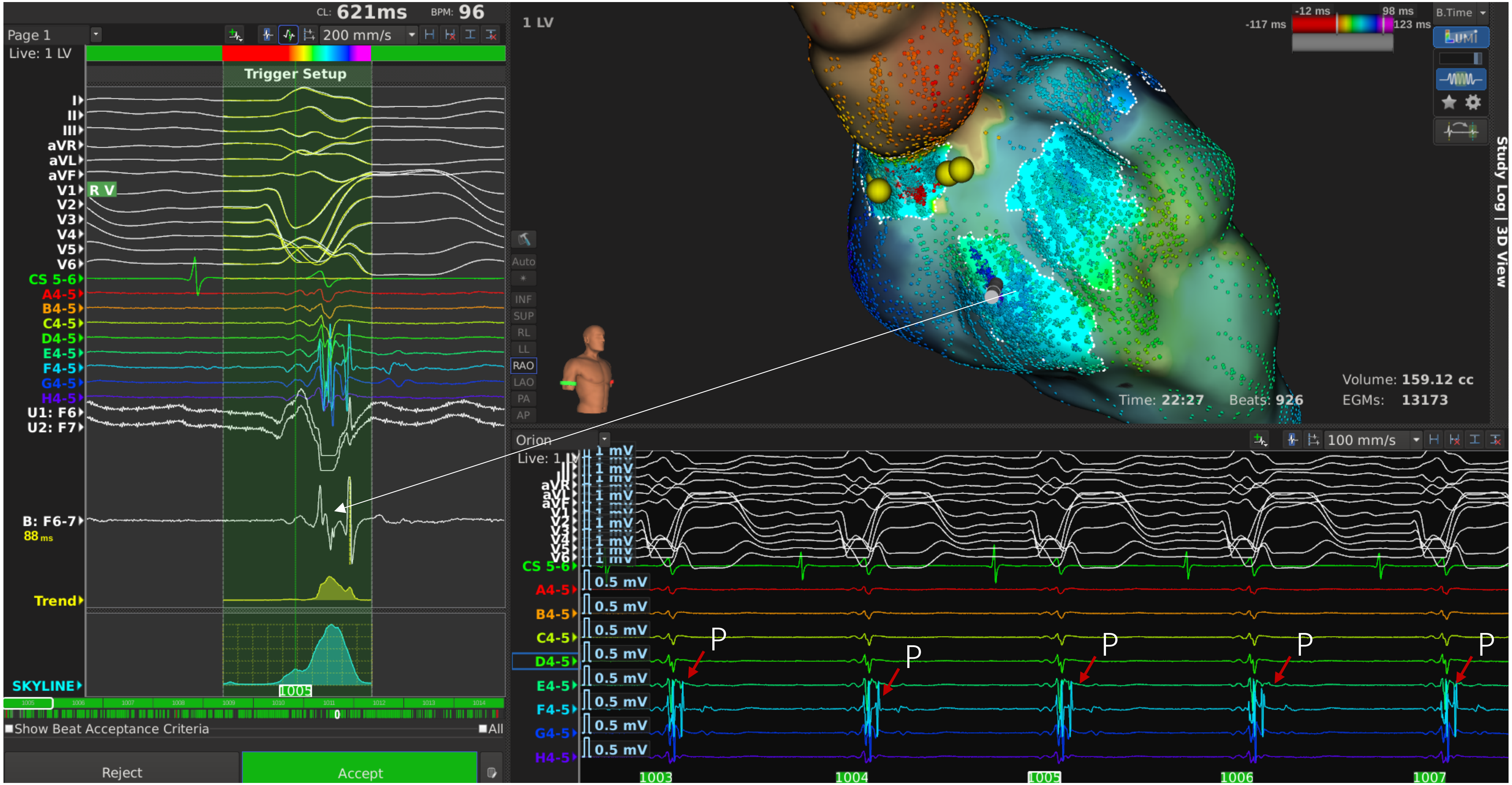

Mechanical stimulation of these parts shows that the left bundle branch potential is reversed, which is earlier than the local ventricular activation, and the surface QRS complex is also narrowed at this time.

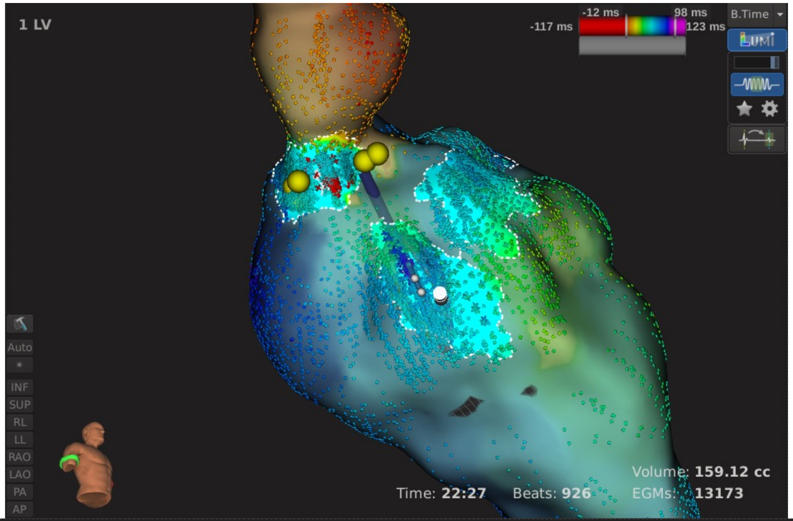

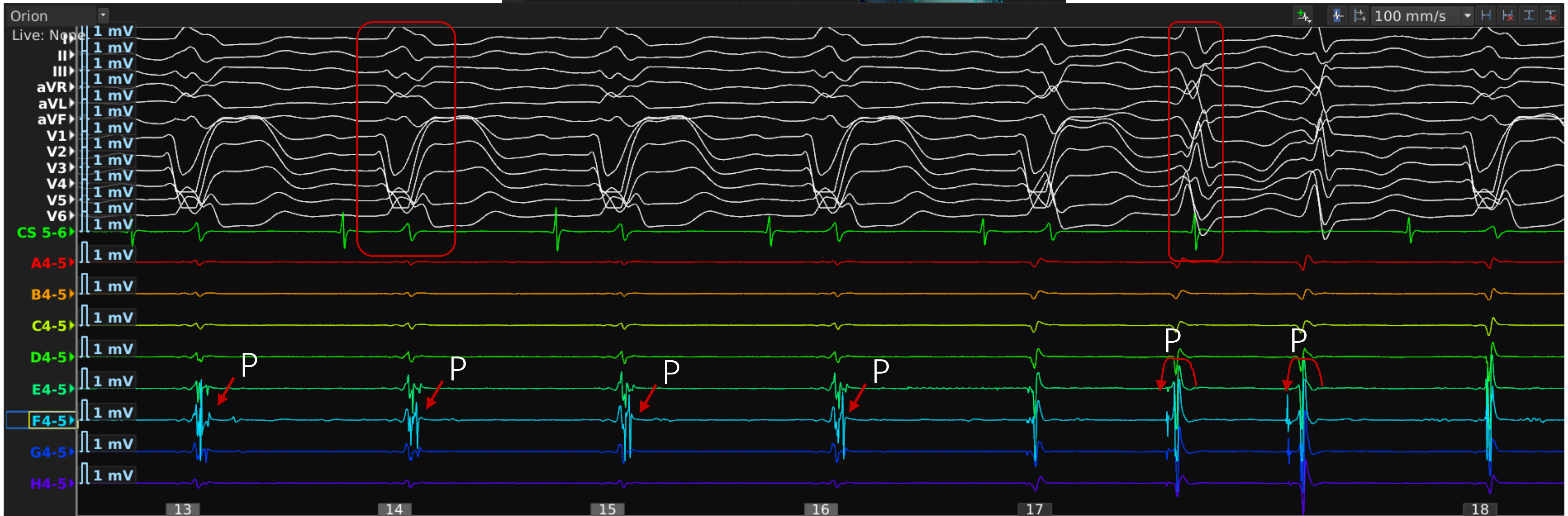

Same as above

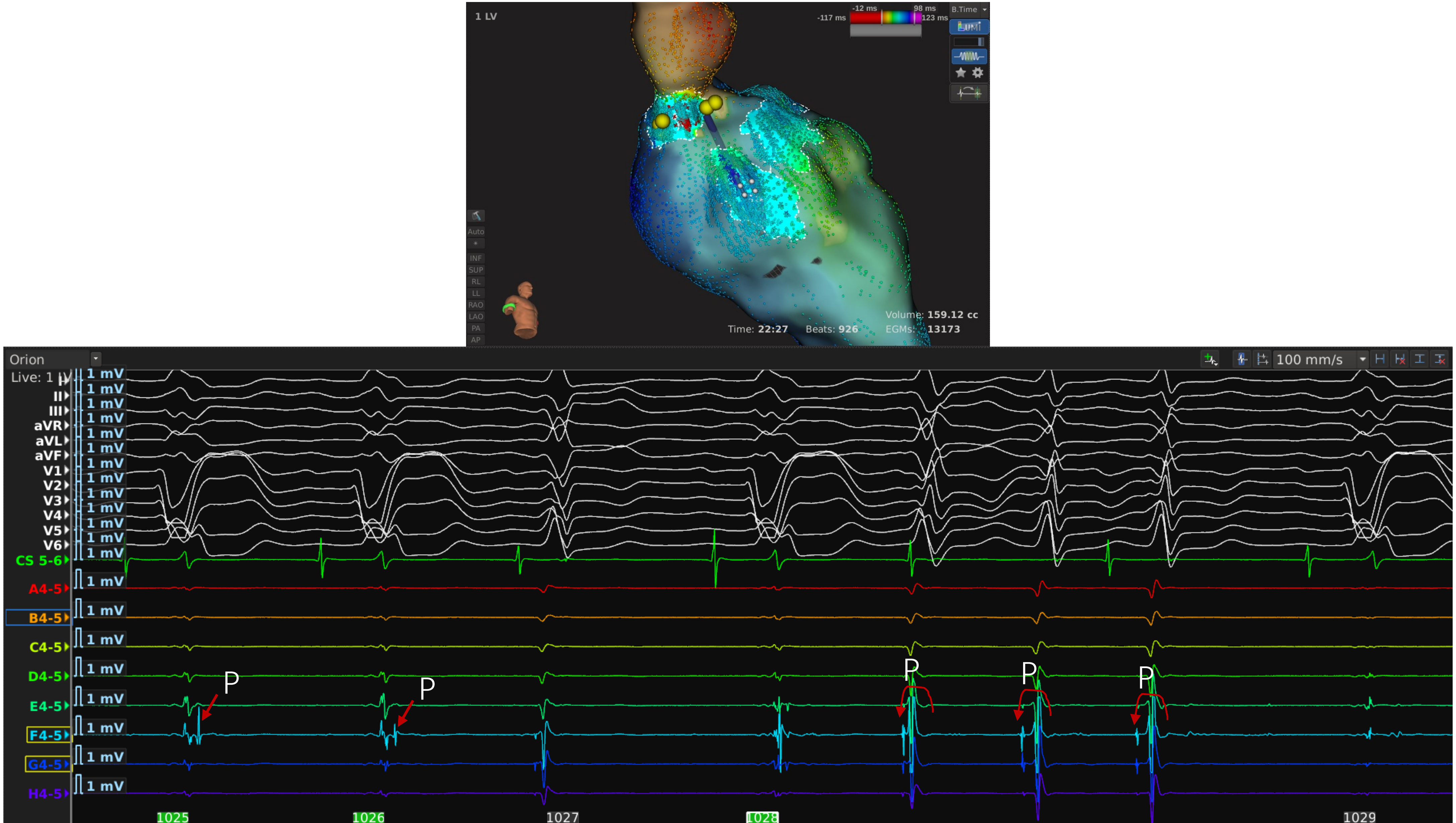

Pacing at this position also narrows the QRS complex

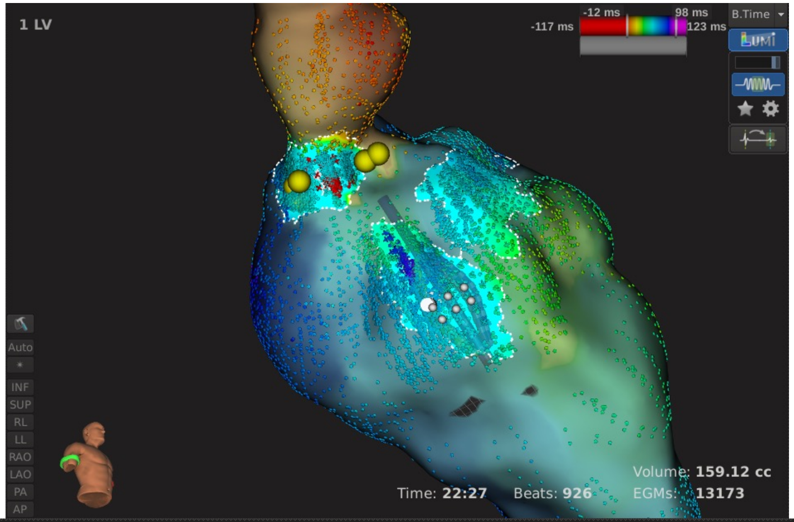

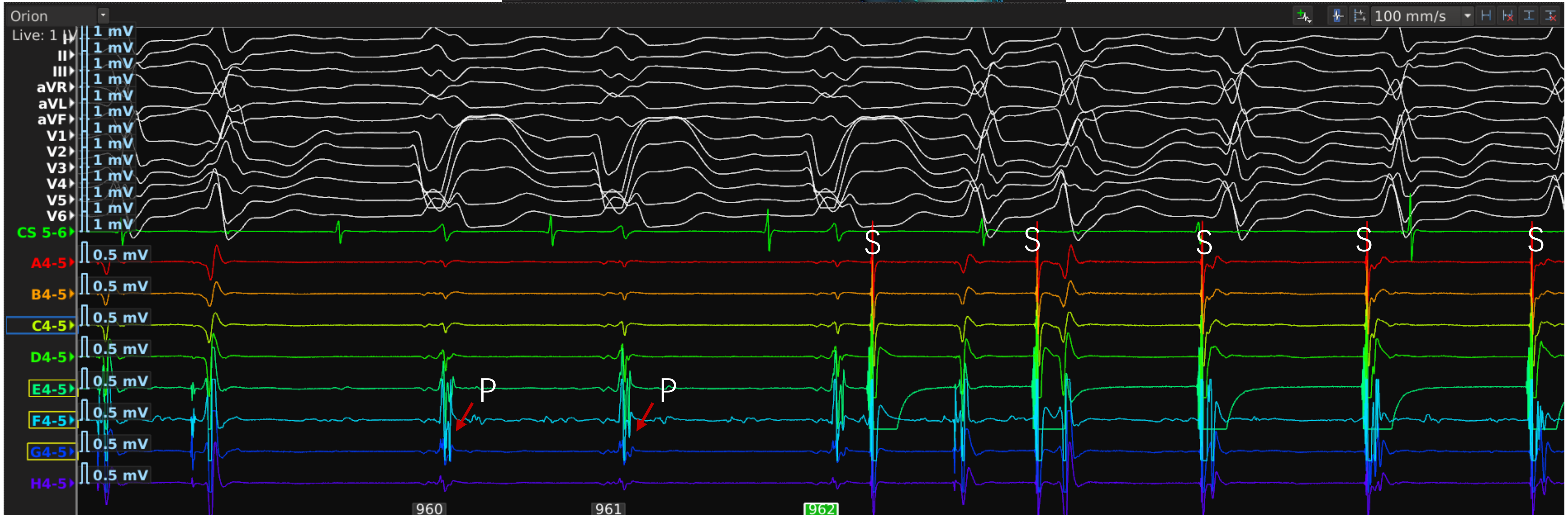

This situation suggests left bundle branch conduction delay, which can be corrected by LBBP pacing, and the location of the mapping can also guide the implantation site of the left bundle branch electrode.
