# Supplementary material for: Three-dimensional electroanatomical mapping guidelines for the selection of pacing site to achieve cardiac resynchronization therapy
Source: Front Cardiovasc Med. 2022 Sep 30;9:843969. doi: 10.3389/fcvm.2022.843969 (PMC9562822; doi:10.3389/fcvm.2022.843969)
Supplement: Supplementary file 2 [file Data_Sheet_2.docx]

**Supplementary Table2. LVEF and synchronization parameters after the operation measured by transthoracic echocardiography analyzed in LBBAP group**

| **LBBAP group (n=42)** |  | |  |  | |  | |
| --- | --- | --- | --- | --- | --- | --- | --- |
|  | LVEF | EA/RR | | | IVMD | | Ts-SD 12 |
| **Mapping (n=25)** |  |  | | |  | |  |
| Baseline | 31.16±8.23 | 0.28 | | | 58.19 | | 145.57 |
| Six month | 37.64±6.18*** | 0.3* | | | 46.38 | | 111.38*** |
| Twelve month | 52.36±6.05*** | 0.34*** | | | 39.9** | | 121.24*** |
| **Non-mapping (n=17)** |  |  | | |  | |  |
| Baseline | 28.24±7.40 | 0.26 | | | 71.62 | | 144.38 |
| Six month | 32.71±6.99*** | 0.28 | | | 69.77 | | 131.62** |
| Twelve month | 40.60±7.84*** | 0.29 | | | 50.23** | | 123.62** |
| *P* value  (mapping vs. non-mapping at 6 month) | 0.009 | 0.488 | | | 0.043 | | 0.007 |
| *P* value  (mapping vs. non-mapping at 12 month) | <0.001 | 0.014 | | | 0.024 | | 0.029 |

Notes. LVEF, left ventricular ejection fraction; EA/RR, EA distance/RR duration; IVMD,interventricular mechanical delay; Ts-SD12, standard deviation of Ts of 12 LV segments. *p<0.05; **p<0.01; ***p<0.001

**Supplementary Table3. LVEF and synchronization parameters after the operation measured by transthoracic echocardiography analyzed in CVP group**

| **CVP group (n=29)** |  |  |  |  |
| --- | --- | --- | --- | --- |
|  | LVEF | EA/RR | IVMD | Ts-SD 12 |
| **Mapping (n=13)** |  |  |  |  |
| Baseline | 30.62±4.25 | 0.27 | 67 | 144.09 |
| Six month | 35,77±5.76* | 0.29 | 55.72* | 130.82** |
| Twelve month | 39.92±5.87** | 0.4** | 52.45* | 127.00** |
| **Non-mapping (n=16)** |  |  |  |  |
| Baseline | 27.31±5.07 | 0.22 | 62.8 | 141.53 |
| Six month | 29.06±4.67** | 0.22 | 59.6** | 137.27 |
| Twelve month | 33.88±6.24*** | 0.23 | 57.53** | 119.73 |
| *P* value  (mapping vs. non-mapping at 6 month) | 0.001 | 0.002 | 0.043 | 0.01 |
| *P* value  (mapping vs. non-mapping at 12 month) | <0.001 | <0.001 | 0.006 | <0.001 |

Notes. LVEF, left ventricular ejection fraction; EA/RR, EA distance/RR duration; IVMD,interventricular mechanical delay; Ts-SD12, standard deviation of Ts of 12 LV segments. *p<0.05; **p<0.01; ***p<0.001
