# Supplementary material for: Three-dimensional electroanatomical mapping guidelines for the selection of pacing site to achieve cardiac resynchronization therapy
Source: Front Cardiovasc Med. 2022 Sep 30;9:843969. doi: 10.3389/fcvm.2022.843969 (PMC9562822; doi:10.3389/fcvm.2022.843969)
Supplement: Supplementary file 3 [file Data_Sheet_3.docx]

**Lead parameters**

The lead parameters, including capture thresholds, pacing impedances, and R-wave amplitudes were in supplementaryat 1week after the implantation. When comparing the LBBAP group with no mapping patients, mapping patients had lower capture thresholds, higher R-wave amplitudes and lower lead impedances, but the differences failed to reach the statistical significance level (capture thresholds: 0.81 ± 0.32 V vs. 0.91 ± 0.35 V, *p* =0.351; R-wave amplitude: 8.55 ± 3.78 ms vs. 7.19± 3.01 ms, *p=* 0.336; lead impedance: 0.71 ± 0.22 kΩ vs. 0.86 ± 0.28 kΩ *p*=0.082). When comparing the CVP group to no mapping patients, mapping patients had higher capture thresholds, higher R-wave amplitudes, but the differences failed to reach the statistical significance level (capture thresholds: 1.10 ± 0.58 V vs. 0.89 ± 0.56 V, *p* =0.378; R-wave amplitude: 8.1 ± 2.79 ms vs. 7.56 ± 6.56 ms, *p* =0.867 ); the lead impedance remained similar in the two subgroups at 1 week after the operation (0.81 ± 0.28 kΩvs. 0.81 ± 0.35kΩ, *p* =0.994).


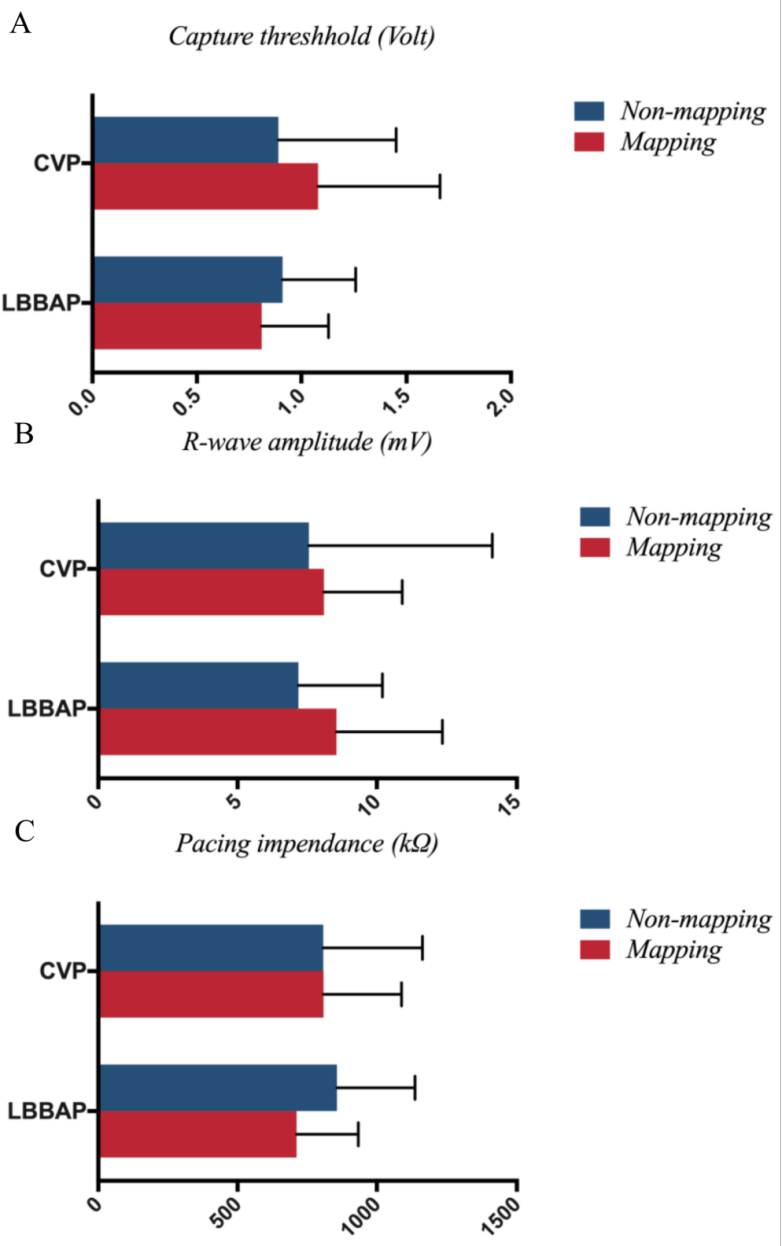
**Supplementary figure**

Supplementary figure 4. Lead parameters in 1 week after the operation. No difference was found between mapping and non-mapping group both in LBBAP and CVP in capture threshold (A), R-wave amplitude (B) nor pacing impedance (C). CVP, coronary venous pacing; LBBAP, Left bundle branch region pacing.
